# A Young Male With Non-cirrhotic Cryptogenic Portal Cavernoma: An Authoritative Case Study

**DOI:** 10.7759/cureus.50570

**Published:** 2023-12-15

**Authors:** Vikram B Vikhe, Devansh Khandol, Aniket A Garud

**Affiliations:** 1 Department of General Medicine, Dr. D. Y. Patil Medical College, Hospital and Research Centre, Pune, IND; 2 Department of Pharmacology, Rasiklal M. Dhariwal Institute of Pharmaceutical Education and Research, Pune, IND

**Keywords:** thrombophilia and cancer screening, rare cause, young male, non-cirrhotic, idiopathic portal cavernoma

## Abstract

The growth of several porto-portal collateral veins encircling an existing stenosed or obstructed entry vein is an uncommon condition known as portal cavernoma. It is traditionally shown as the entry vein thrombosis (portal vein thrombosis - PVT) outcome. A male of 25 years with stomach discomfort for three days that was acute, nonprogressive, and was not accompanied by fever, loose stools, or vomiting. After he had undergone abdominal ultrasonography, portal vein thrombosis was discovered, and based on no involvement of suprahepatic veins according to ultrasonography, Budd-Chiari syndrome was ruled out. It was accompanied by dilated periportal tortuous veins and visible mesenteric and peri-splenic collaterals. Moderate splenomegaly was also present. All these features on ultrasound were suggestive of the "portal cavernoma" formation. The patient is not an alcoholic and does not have any chronic, hereditary, or metabolic liver disease. Thrombophilia and cancer screening through tumor markers were also negative. We, with this, present a rare case of non-cirrhotic idiopathic portal cavernoma. This rare case contributes to advancing medical and scientific knowledge that will encourage further dialogue on the topic.

## Introduction

Portal cavernoma is common in people who have cirrhotic liver disease, liver or biliary neoplasm, and hematologic or gastrointestinal disorders. In individuals who are not cirrhotic, cryptogenic portal vein thrombosis (PVT) is rare, with only around 25% of cases having an unidentified cause. Patients typically present with ascites, and splenomegaly, and may have severe symptoms of either rectal or oesophageal variceal bleed [1]. If the condition is not addressed, it can lead to intestinal ischemia, septic thrombosis, varices, portal hypertension, and portal vein cavernous transformation. Currently, there are no standardised guidelines or established best practices for addressing this medical condition in cirrhotic and non-cirrhotic persons (which can be easily differentiated based on the history of any addiction or the presence of any risk factors, none of which were present in our patient, which is described below in case presentation). According to the Baveno and Sarin classification of portal thrombosis, our patient has a case of chronic PVT. Therefore, the most effective approach involves close monitoring for potential complications and timely follow-up care.

## Case presentation

A 25-year-old male, a labourer from a lower socioeconomic status group, presented to the physician with complaints of abdominal pain for three days, which was sudden and acute in onset, mild nonprogressive diffuse in nature, with no variation with meals, no relieving nor aggravating factors, not associated with loose stools, nauseating sensation, high temperature, dark-coloured stool, and blood in the vomitus. He had no history of similar complaints. No history of any comorbidities like tuberculosis or diabetes/blood transfusion in the past. No recent vaccination history. There is no history of any addiction to alcohol, tobacco, or smoking. No history of previous hospitalisation. No history of anemia or jaundice in the past. On examination, the patient was** **afebrile, with normal vitals. There was no history of schistosomiasis. No signs of liver cirrhosis stigmata present with no abnormal general examination findings. On a per-abdomen examination, the abdomen was soft, non-tender on palpation. No guarding/rigidity was present. No ascites/dilated veins were seen. The spleen was palpable 2 cm below the costal margin. There was no hepatomegaly.** **ECG showed normal sinus rhythm. Normal heart sounds were heard on auscultation. No murmur was heard. Respiratory and central nervous system examinations were within normal limits.

**Figure 1:**
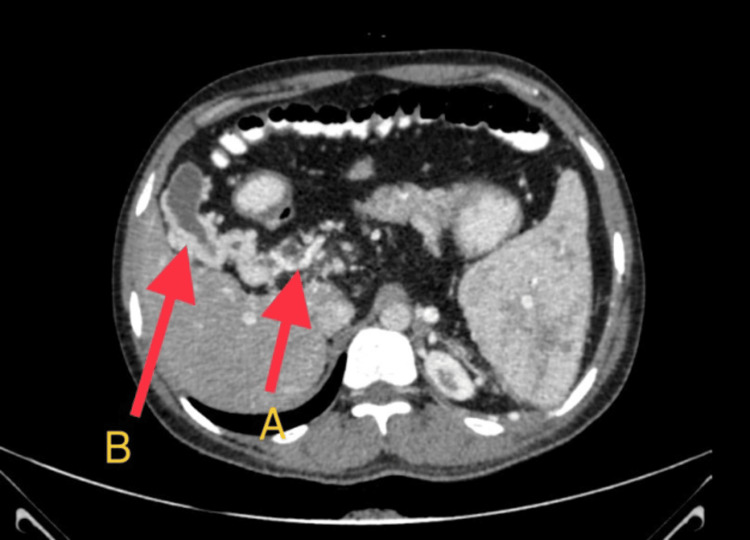
An axial section of the CECT abdomen showing multiple dilated collateral vessels (red arrow A) causing compression of the distal column of CBD (red arrow B).

**Figure 2:**
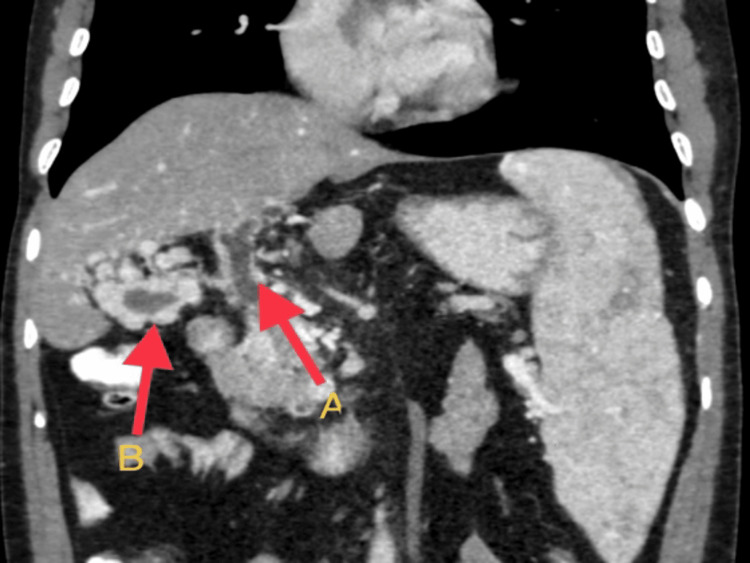
This coronal section of the CECT abdomen shows a portal cavernoma formation (red arrow B) with upstream dilatation of distal CBD (red arrow A).

Routine laboratory tests including hemogram (hemoglobin of 13.5 g/dL, WBC of 5,500/mm^3^, platelet of 170,000/mm^3^) and others such as liver (total bilirubin of 0.8, direct bilirubin of 0.5, indirect bilirubin of 0.3, SGOT of 47, SGPT of 32, and ALP of 80) and renal function tests (urea of 32 and creatinine of 0.6), along with fasting lipid levels (total cholesterol of 180 mg/dL, triglycerides of 110 mg/dL, LDL cholesterol of 98 mg/dL, and HDL cholesterol of 50 mg/dL) were normal. The thrombophilia profile, antinuclear antibody blot, direct and indirect Coomb's test, tumor markers, and viral markers, including CMV, EBV, HAV, HBV, HCV, HEV, and HIV, were all negative. There were no laboratory parameters suggestive of JAK STAT-2 mutation or paroxysomal nocturnal hemoglobinuria as all such reports came negative. A fibroscan was done that showed a liver thickness of 8.0k Pa. The 2D echocardiography was normal. Ultrasonography of the abdomen was suggestive of a shrunken liver with coarse echotexture. Tortuous portal veins with multiple periportal, mesenteric, and perisplenic collaterals were present. There was gallbladder wall edema with mild splenomegaly. CECT (contrast-enhanced computed tomography) abdomen and pelvis were suggestive of a small size of the liver with marked atrophy of the left lobe with normal attenuation. The portal vein was not visualised, and, instead, multiple dilated collateral vessels were noted in the portocaval and lineorenal region. The collaterals appeared to be compressing on a distal column of CBD leading to upstream dilatation of CBD and central intrahepatic biliary radicles (as seen in Figures 1-2 with multiple red arrows). Mild splenomegaly was also present.

**Figure 3:**
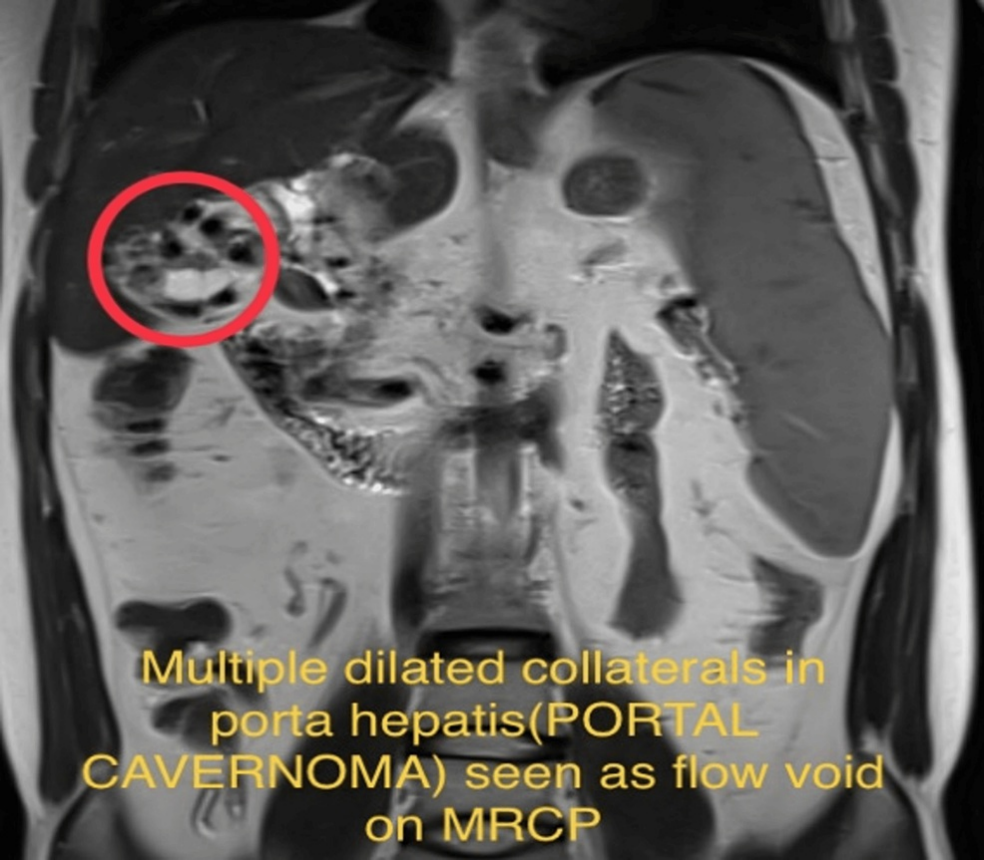
This coronal section of magnetic resonance cholangiopancreatography (MRCP) shows portal cavernoma considered as flow void in Figure 3 with a red circle.

**Figure 4:**
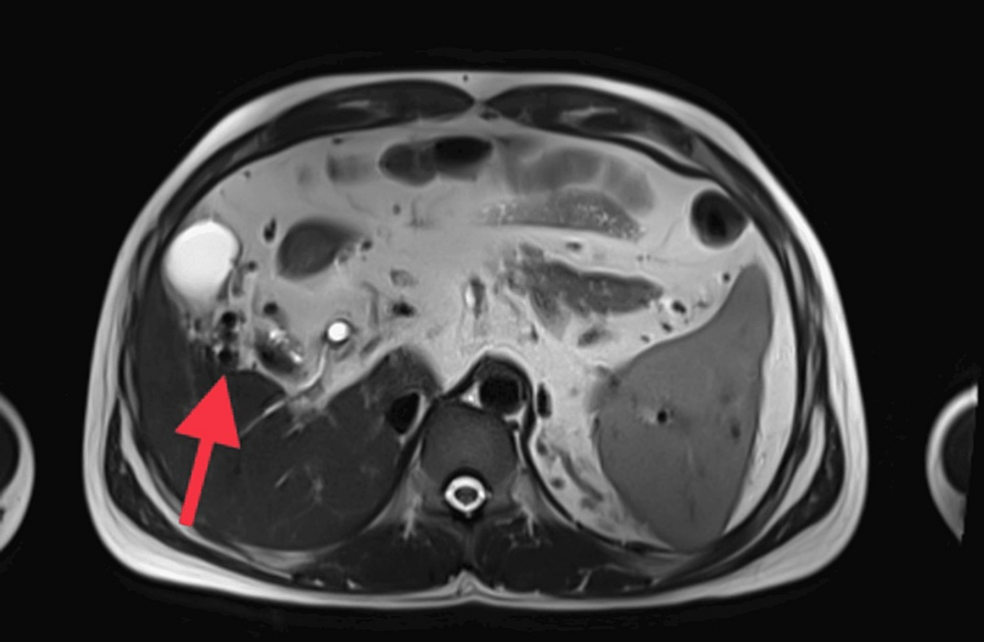
Dilated collaterals as seen with a red arrow in this axial section of MCRP.

Magnetic resonance cholangiopancreatography (MRCP) showed a small size of the liver (9.5 cm) with marked atrophy of the left lobe. The portal vein was not distinctly visualised instead multiple dilated collateral vessels were noted in porta hepatis, pericholecystic region, peripancreatic region, perisplenic region, splenic hilum region, linorenal region (as seen in Figure 3 with red circle and in Figure 4 with arrow). These collaterals appeared to be compressing on the distal column of CBD, leading to upstream dilatation of CBD and central intrahepatic biliary radicles. Intrahepatic biliary radicles were dilated. The spleen was enlarged in size with a craniocaudal extent of 17 cm. Multiple well-defined hypointense lesions were noted. They were scattered in the entire spleen. These are Gamna-Gandy bodies - hemosiderin deposits due to portal hypertension.

An upper gastrointestinal endoscopy of the patient was done, which presented three large columns of esophageal varices, and an esophageal variceal band ligation (EVL) was done for the same. Moreover, it shows that the patient has portal hypertensive gastropathy (PHG) and duodenopathy (PHD), along with gastric antral vascular ectasia (GAVE). Following endoscopy, the patient was started on tablet carvedilol 3.125 mg once a day. A repeat endoscopy was done after three weeks, which showed a significant reduction in varices with only small esophageal varices present, with a resolution of portal hypertensive duodenopathy (PHD), while PHG and GAVE were still present. The patient is asymptomatic at present. He is on antioxidants and a tablet of carvedilol 3.125 mg once a day. He is regularly coming for follow-ups once monthly.

## Discussion

A procedure that causes clots to form inside of vessels is called thrombosis. For clot configuration, there exist three fundamental groups of components, which can be referred to as Virchow's group of three. The three factors are blood stasis, cellular damage, and hypercoagulability. The vicinity of a coagulation tangle, such as factor V Leiden, is referred to as hypercoagulability. During a hypercoagulability screen, prothrombin quality change, homocysteine, protein C and S, lupus anticoagulant, factor V Leiden mutation, antithrombin III, and coagulation parameters such as prothrombin time-international normalised ratio (PT-INR), activated partial thromboplastin time (APTT), fibrinogen, and platelets are routinely tested for. The disruption of normal blood flow in a vessel is referred to as blood stasis. Endothelial or cellular injury is caused by damage to vessels, like from procedures and unintentional wounds. As a result, monitoring and treatment of thrombotic events are coordinated not only to essentially stop thrombi from moving but also to identify potential fundamental reasons to avoid similar incidents in the future.

The incidence of PVT ranges from patients discovered by accident during imaging to symptomatic cases where fevers and stomach pain are the most frequent side effects. While entrance hypertension does not manifest clinically in and of itself, patients frequently present with associated sequelae. Entrance hypertension is another typical complication noticed with persistent PVT. One of the causes of PVT is paroxysmal nocturnal hemoglobinuria (PNH) [2]. 40.7% and 10.2% events of thrombosis in PNH have been reported to be impacted by thrombosis occurring in the portal [3] and periportal veins, respectively. Paroxysmal nocturnal hemoglobinuria along with PVT has a very high mortality rate of 60-70% [4]. JAK-2 mutation is another rare risk factor. Occult malignancy is the cause of PVT. It has been established that hepatitis, cytomegalovirus (CMV) [5], or Epstein-Barr infection increases the risk of splanchnic vascular thrombosis, and, thereby, should be kept as a rare cause for PVT [6]. There has been an instance of [7] portal cavernoma caused by CMV.

**Figure 5:**
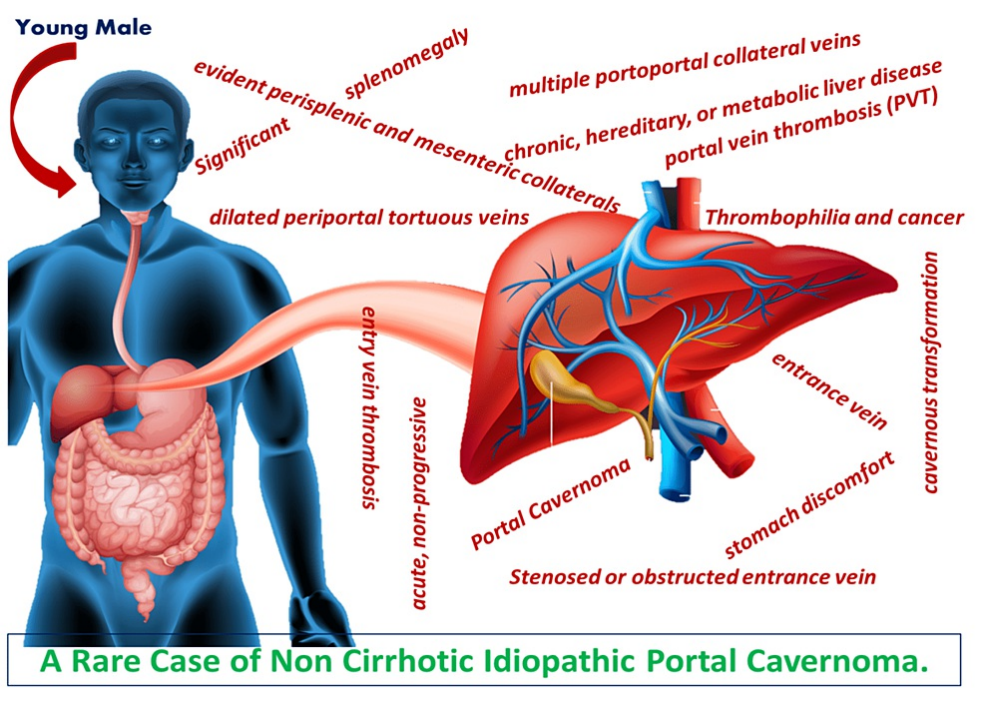
Figure depicts a graphical (animated) representation of our patient clearly describing all features, which he had, thus supporting his diagnosis of idiopathic portal cavernoma. Image credits: Dr. Devansh Khandol

Researchers have estimated that the capacity to identify cirrhosis with ultrasound imaging is 78.7% to 82.2% effective and with computed tomography, 77.1% [8]. Early anticoagulation has helped reduce disease progression and, thus, complications as per data with increased chances of recanalisation portal vein [9] and thereby reduced mortality [10,11]. Complications include portal hypertension causing variceal bleeding because of shunting between systemic and portal circulation [12] and local intestinal necrosis both of which are devastating. Rarely abdominal distension and splenomegaly can occur. Different studies demonstrate different follow-up/review periods some at 12 months or longer, some at less than 12 months, and some between zero and six. Ultimately, the choice is made based on each case individually, considering the disease's course and symptom development. Figure 5 shows a pictorial representation of our patient with portal cavernoma and the symptoms and complications that he had.

## Conclusions

After portal cavernoma is brought on by cirrhosis, gastrointestinal infections, neoplasms, hypercoagulable illnesses, or surgery, the portal vein typically undergoes a cavernous transformation. All of the criteria listed above did not apply to our patient. We, thus, report a unique instance of idiopathic portal vein cavernous change. Continuous monitoring and follow-up are the only possible care that we can give to this patient because of the lack of clear-cut guidelines.

## References

[1] Sogaard KK, Astrup LB, Vilstrup H, Gronbaek H (2007). Portal vein thrombosis; risk factors, clinical presentation and treatment. BMC Gastroenterol.

[2] Van Bijnen ST, Van Heerde WL, Muus P (2012). Mechanisms and clinical implications of thrombosis in paroxysmal nocturnal hemoglobinuria. J Thromb Haemost.

[3] Qi X, He C, Han G (2013). Prevalence of paroxysmal nocturnal hemoglobinuria in Chinese patients with Budd-Chiari syndrome or portal vein thrombosis. J Gastroenterol Hepatol.

[4] Salvagno GL, Pavan C, Lippi G (2018). Rare thrombophilic conditions. Ann Transl Med.

[5] Rao R, Grosel J (2018). Acute portal vein thrombosis in a 59-year-old male with JAK2 V617F mutation. Radiol Case Rep.

[6] Galli L, Gerdes VE, Guasti L, Squizzato A (2014). Thrombosis associated with viral hepatitis. J Clin Transl Hepatol.

[7] Burkey C, Teng C, Hussein KI, Sabetta J (2020). Cytomegalovirus (CMV)-associated portal vein thrombosis in a healthy, immunocompetent man. BMJ Case Rep.

[8] Sharma S, Khalili K, Nguyen GC (2014). Non-invasive diagnosis of advanced fibrosis and cirrhosis. World J Gastroenterol.

[9] Choudhry AJ, Baghdadi YM, Amr MA, Alzghari MJ, Jenkins DH, Zielinski MD (2016). Pylephlebitis: a review of 95 cases. J Gastrointest Surg.

[10] Plessier A, Darwish-Murad S, Hernandez-Guerra M (2010). Acute portal vein thrombosis unrelated to cirrhosis: a prospective multicenter follow-up study. Hepatology.

[11] Hall TC, Garcea G, Metcalfe M, Bilku D, Dennison AR (2011). Management of acute non-cirrhotic and non-malignant portal vein thrombosis: a systematic review. World J Surg.

[12] Trebicka J, Strassburg CP (2014). Etiology and complications of portal vein thrombosis. Viszeralmedizin.

